# Ba_3_[Sn(OH)_6_][SeO_4_]_2_·3H_2_O, a hydrated 1:2 double salt of barium hexa­hydroxidostannate(IV) and barium selenate(VI)

**DOI:** 10.1107/S2056989022007198

**Published:** 2022-07-19

**Authors:** Hans Reuter, Shouassi Kamaha

**Affiliations:** a Chemistry, Osnabrück University, Barabarstr. 7, 49069 Osnabrück, Germany; University of Kentucky, USA

**Keywords:** crystal structure, double salt, hexa­hydroxidostannate(IV), selenate(VI), primary building units, secondary building units

## Abstract

Single crystals of tribarium hexa­hydroxidostannate(IV) bis­[selenate(VI)] trihydrate, Ba_3_H_12_O_17_Se_2_Sn or Ba_3_[Sn(OH)_6_][SeO_4_]_2_·3H_2_O, prepared from solid BaSnO_3_ and aqueous Na_2_[SeO_4_] solutions have hexa­gonal (*P*6_3_) symmetry. The structure consists of four different primary building units: a hexa­hydroxidostannate(IV) ion, two different selenate(VI) ions, all three of point group symmetry *C*_3_, and a mono-capped {BaO_9_}-square anti­prism of point group symmetry *C*_1_. The secondary building units result from three of the barium coordination polyhedra linked together *via* common edges. While one of the two tetra­hedral voids formed from these trimeric units is filled by one bidentate, chelating μ_2_-selenate ion, the other one remains unoccupied as the corresponding second selenate ion only acts as a monodentate, μ_1_-ligand. SBUs are completed by hexa­hydroxidostannate(IV) ions sharing adjacent edges on the uncapped faces of the three, mono-capped square anti­prisms. These SBUs are arranged into layers *via* common edges on the uncapped, square faces of the {BaO_9_} coordination polyhedra in a way that the hexa­hydroxidostannate(IV) ions act as linkage between two neighboring layers.

## Abstract 2

The title compound, Ba_3_[Sn(OH)_6_][SeO_4_]_2_·3H_2_O, was prepared by mimicking geochemical crystallization processes, and the hexa­gonal crystal structure determined. Its hierarchical structure comes from four different primary building units, setting up secondary building units that are arranged into layers *via* their {BaO_9_} coordination polyhedra and cross-linked with the SBUs of adjacent layers *via* common [Sn(OH)_6_]^2−^ ions.

## 1. Chemical context

The hexa­hydroxidostannate(IV) ion, [Sn(OH)_6_]^2−^, is a well established tin(IV) anion in chemistry (Scholder, 1981 ▸), mineralogy (Strunz & Nickel, 1998 ▸) and even archaeology (Basciano *et al.* 1998 ▸), although the number of well defined and structurally described compounds is rather low, especially in case of two-valent cations as these compounds are only slightly soluble. In a former paper (Kamaha & Reuter, 2009 ▸), we demonstrated strategies for how to circumvent these difficulties by combining slow anion formation with slow crystallization, mimicking to some extent geochemical crystal formation processes.

Here we present our results on experiments where we offered selenate(VI) anions parallel to the slow formation of hexa­hydroxidostannate(IV) ions, as possible co-anions during crystallization. In a typical experiment we exposed BaSnO_3_ pellets to a Na_2_SeO_4_-solution, resulting after a long period in the formation of colorless, hexa­gonal prisms of Ba_3_[Sn(OH)_6_][SeO_4_]_2_·3H_2_O, a hydrated 1:2 double salt of barium hexa­hydroxidostannate(IV), Ba[Sn(OH)_6_], and barium selenate(VI), Ba[SeO_4_]. From both compounds, only the structure of the selenate has been described in the literature (Andara *et al.*, 2005 ▸).

## 2. Structural commentary

The title compound crystallizes in the non-centrosymmetric, hexa­gonal space group *P*6_3_ and was refined as an inversion twin, giving a Flack parameter of 0.037 (11). With two formula units in the unit cell, the asymmetric unit consists of 1/3 formula unit: a Ba^2+^ ion and a water mol­ecule, both in general positions, and two crystallographically independent [SeO_4_]^2−^ ions and one [Sn(OH)_6_]^2−^ ion, all three having the point group *C*_3_. In addition to the {BaO_9_}-coordination polyhedron, these complex anions represent the primary building units, PBUs.

**Figure 1:**
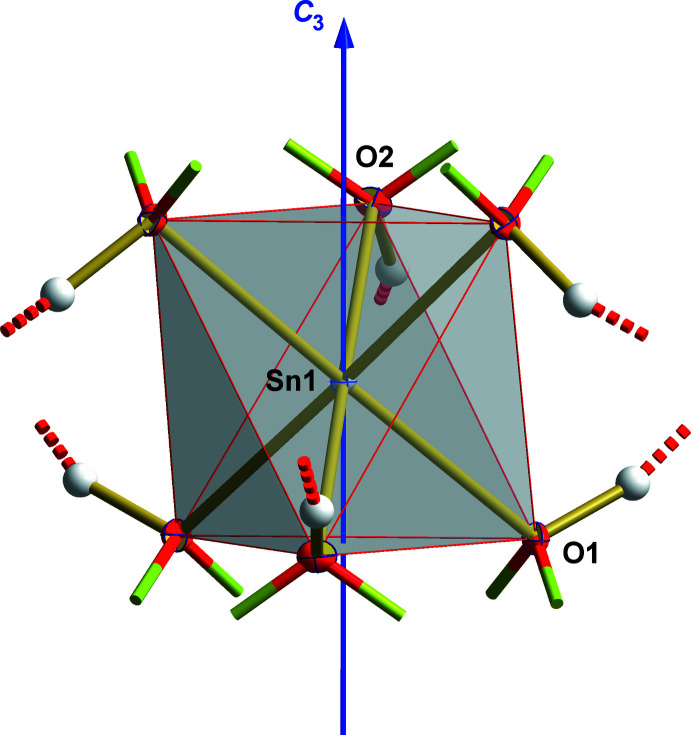
Ball-and-stick model of the [Sn(OH)_6_]^2−^ ion with atom numbering of the asymmetric unit and orientation of the threefold rotation axis (blue). With exception of the hydrogen atoms, which are shown as spheres of arbitrary radius, all other atoms are drawn as displacement ellipsoids at the 40% level. Bonds between oxygen and barium are indicated as shortened, two-colored sticks while hydrogen bonds are visualized as dashed lines (red).

The two crystallographically different Sn–O distances within the hexa­hydroxidostannate(IV) anion (Fig. 1 ▸, Table 1 ▸) are identical within standard deviations [*d*(Sn1—O1) = 2.052 (2) Å and *d*(Sn1—O2) = 2.054 (2) Å]. The mean value of 2.053 (2) Å is somewhat shorter than the mean value of 2.060 (10) Å observed in other hexa­hydroxidostannates (Kamaha & Reuter, 2009 ▸), but lies in the observed range of 2.039–2.075 Å. Deviations from the geometry of a regular octa­hedron are better expressed in terms of the bond angles, best described by the non-linearity of the octa­hedron axes, which show bond angles of 178.7 (1)°. All oxygen atoms of the [Sn(OH)_6_]^2−^ ion coordinate to two different Ba atoms in a μ_2_-coordination mode, while the hydrogen atoms are involved in hydrogen bonds (Table 2 ▸) with the oxygen atoms O3 and O7 of the two different [SeO_4_]^2−^ ions.

**Figure 2:**
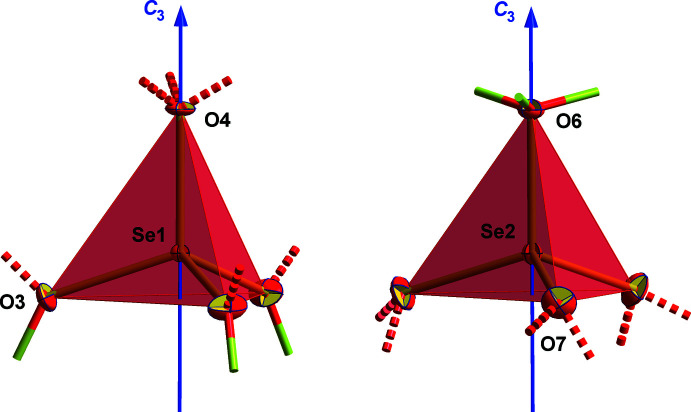
Ball-and-stick models of the two crystallographically independent [SeO_4_]^2−^ ions with atom numbering of the asymmetric unit and orientation of the threefold rotation axis (blue). With exception of the hydrogen atoms, which are shown as spheres of arbitrary radius, all other atoms are drawn as displacement ellipsoids at the 40% level. Coordination bonds between oxygen and barium are drawn as shortened, two-colored sticks, hydrogen bonds between oxygen and hydrogen atoms of water mol­ecules and hydroxyl groups are drawn as dashed lines (red).

As a result of their *C*_3_ symmetry, the structural parameters (Table 3 ▸) of both selenate(VI) ions are restricted to those between the two crystallographically different oxygen atoms as one (denoted *apical* in the following) of them is situated on the threefold rotation axis (O4 in the first selenate, O6 in the second selenate) while the others (hereafter *basal*) (O3/O7) are in general positions. While the mean value of 1.638 (8) Å over all eight Se—O bond lengths is in excellent agreement with the mean Se—O bond lengths in other selenates [neutron data: *d*(Se—O) = 1.641 Å, Mg[SeO_4_]·7H_2_O, *T* = 10 K (Fortes & Gutmann, 2014 ▸); *d*(Se—O) = 1.637 Å, Mg[SeO_4_] · 9H_2_O, *T* = 100 K (Fortes *et al.* 2015 ▸); X-ray data: *d*(Se—O) = 1.639 Å, Na_2_[SeO_4_]·1.5H_2_O and Na_2_[SeO_4_]·10H_2_O, *T* = 100 K (Weil & Bonneau, 2014 ▸), *d*(Se—O) = 1.639 Å, Mg[SeO_4_]·6H_2_O, *T* = 293 K (Kolitsch, 2002 ▸)] the individual Se—O distances differ significantly, reflecting the different functionality of both kind of oxygen atoms. Bonds to the *apical* oxygen atoms are considerably longer [1.654 (4), 1.648 (4) Å] than those to the *basal* ones [1.634 (2), 1.633 (2) Å]. In the first selenate ion, the *apical* oxygen atom acts as acceptor of three hydrogen bonds, while the corresponding oxygen atom of the second selenate ion coordinates to three barium ions. On the other hand, the three *basal* oxygen atoms act as acceptor of one hydrogen bond and also perform coordinative bonds, each to a different barium ion, in the first selenate ion while those of the second selenate ion accept two hydrogen bonds. Besides bond-length differences, deviations from the geometry of a regular tetra­hedron result in both selenate ions having bond angles widening between the *basal* oxygen atoms, giving them the shape of slightly flattened trigonal pyramids rather than strict tetra­hedra (Fig. 2 ▸).

**Figure 3:**
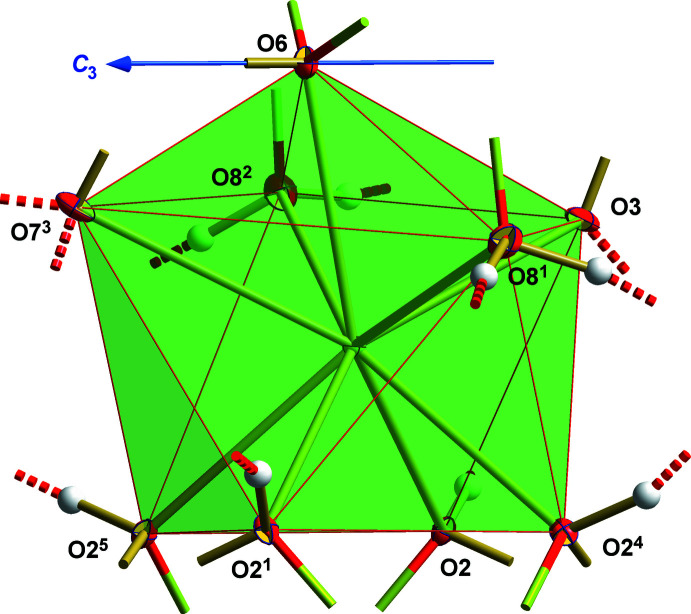
Ball-and-stick model of the mono-capped, square-prismatic {BaO_9_} coordination polyhedron. Atom colors and bond design as in Fig. 2 ▸. Symmetry codes: (1) *y*, 1 − *x* + *y*, 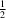 + *z*; (2) 2 − *x*, 1 − *y*, 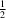 + *z*; (3) 1 − *y*, *x* − *y*, *z*; (4) 1 − *x* + *y*, 2 − *x*, *z*; (5) 1 + *x* − *y*, *x*, 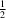 + *z*.

The coordination sphere of the barium ion consists of nine oxygen atoms: two from water mol­ecules, four from two [Sn(OH)_6_]^2−^ ions, one from the first selenate ion and two, respectively, from the second selenate ion (Fig. 3 ▸). In summary, this {Ba(μ_2_-OH)_4_(H_2_O)_2_(μ_2_-O_Se2_)_2_(μ_1_-O_Se1_)} coord­ination sphere has the shape of a mono-capped square anti­prism. The uncapped face of this coordination polyhedron only is built up from the oxygen atoms of two hexa­hydroxidostannate ions. Its shape is almost square [maximal angle deviations from rectangular: ±0.6 (1)°, maximal deviation from planarity: ±0.0132 Å, side lengths: 2.7705 (2)–3.1720 (2) Å]. In contrast, the capped face of the square anti­prism consists of oxygen atoms from two water mol­ecules and from *basal* oxygen atoms of the two different selenate ions. Its shape [maximal deviation from planarity: ±0.0022 (2) Å] is much better described as an acute trapezoid with a longer/shorter base of 4.4606 (2)/3.7164 (2) Å, legs of 3.4108 (2)/3.2331 (2) Å and angles between 103.01 (1) and 78.42 (1)°. The dihedral angle between these planes is 5.64 (1)°. These deviations from a regular square anti­prism are mainly caused by coordination to the selenate ions, as the *apical* oxygen atom of the second one constitutes the cap of the {BaO_9_} coordination polyhedron, giving rise to a bidentate-chelating coordination mode of this selenate ion while the first selenate ion only acts as monodentate ligand. Ba—O bond lengths (Table 3 ▸) range from 2.715 (2) to 3.106 (3) Å, mean value 2.837 Å. Bonds between the barium ion and the oxygen atoms of the hexa­hydroxidostannate ions are of comparable lengths [d(Ba-O1/O2) = 2.737 (2)–2.782 (2) Å] as are those between the barium ion and the water mol­ecules [2.880 (2)/2.931 (2) Å]. The longest bond [*d*(Ba—O7) = 3.106 (3) Å] is between the barium ion and the *basal* oxygen atom of the second selenate ion, while the shortest one [*d*(Ba—O3) = 2.715 (2) Å] leads to the *basal* atom of the first selenate ion.

## 3. Supra­molecular features

**Figure 4:**
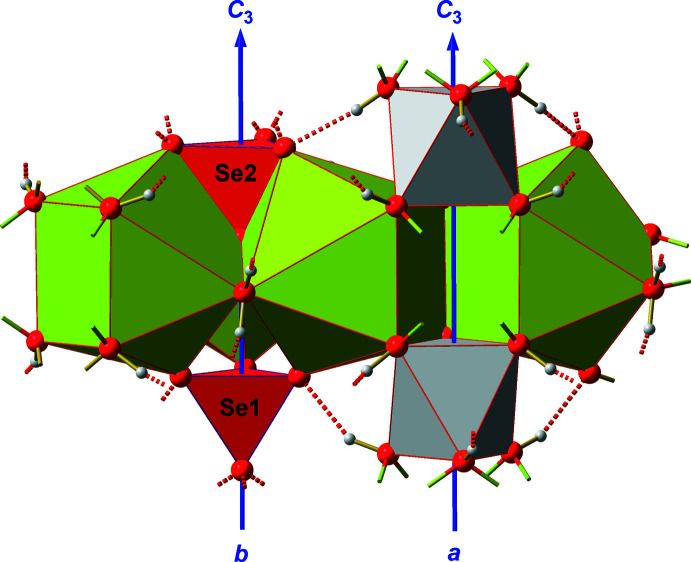
Polyhedral model showing the inter­connection of the PBUs (selenate ions in red, {BaO_9_} coordination polyhedra in green, [Sn(OH)_6_]^2−^ ions in gray) and their orientation with respect to the different threefold rotation axes (blue, letter = Wyckoff position) of space group *P*6_3_. Oxygen atoms (red) and hydrogen atoms (gray) are drawn as spheres of arbitrary radius. Hydrogen bonds are indicated as broken red sticks, visible Ba—O coordinative bonds as shortened, two-colored sticks, and visible Sn—O bonds as shortened, brass-colored sticks. In order to show the linkage of the SBUs and to visualize the trigonal–prismatic void between the hexa­hydroxidostannate ions, one additional {BaO_9_} coordination polyhedron and one additional [Sn(OH)_6_]^2−^ ion are also shown.

The inter­action of the four different PBUs is visualized in Fig. 4 ▸. The most prominent part of the resulting secondary building units, SBUs, consists of three {BaO_9_}-coordination polyhedra related to each other *via* the threefold rotation axes in Wyckoff position *b.* These three PBUs are linked together *via* common edges, each of them composed of the coordinated water mol­ecule and the *apical* oxygen atom of the second selenate ion. In addition, this selenate ion shares its remaining three, *basal* oxygen atoms with the three surrounding barium ions, thus filling the tetra­hedral void between the three {BaO_9_} coordination polyhedra. On the other hand, the opposite void of the trimeric {BaO_9_} unit is empty, as the first selenate ion only shares its three *basal* oxygen atoms with the three {BaO_9_} coordination polyhedra but not the *apical* one. Each SBU is completed by a hexa­hydroxidostannate(IV) ion sharing one edge with the uncapped face of the mono-capped {BaO_9_} anti­prisms.

These secondary building units are linked together with three others, each *via* a common edge of the uncapped square of the {BaO_9_} coordination polyhedra. In this way, each {BaO_9_} coordination polyhedron shares two opposite edges of its square faces with two neighboring barium coordination polyhedra, resulting in a trigonal–prismatic void between the three inter­connected SBUs. All corners of these voids consist of hydroxyl groups from [Sn(OH)_6_]^2−^ ions with the tin atoms of these PBUs situated on threefold rotation axes in Wyckoff position *a*.

**Figure 5:**
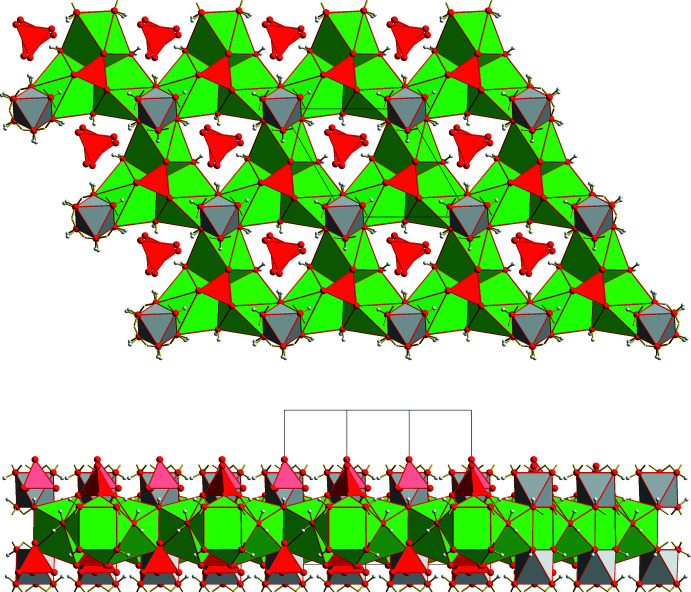
Polyhedral model showing the aufbau principle of the layers (top view above, side view below) as result of the inter­connection of the SBUs. Polyhedra colors according to Fig. 1 ▸ to 3. Isolated selenate ions (Se2) in the three-sided pores belong to adjacent layers. The corresponding hydrogen bonds are omitted for clarity.

In summary, the SBUs are arranged in layers perpendicular to the *c* axis direction (Fig. 5 ▸). The pores within these layers are occupied by selenate ions (Se2) of adjacent layers. These selenate ions are connected with the layer *via* hydrogen bonds (Table 2 ▸) to the water mol­ecules and hydroxyl groups of the [Sn(OH)_6_]^2−^ ions, while the latter cross-link adjacent layers.

**Figure 6:**
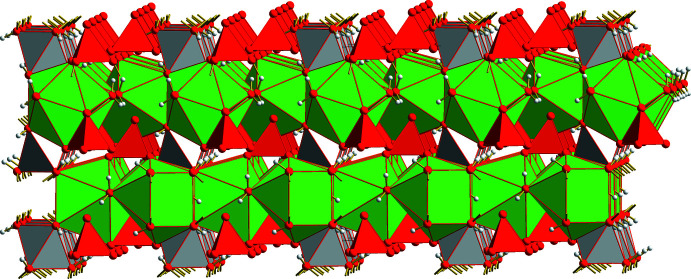
Polyhedral model showing the packing of two layers.

Adjacent layers are rotated through 120° against each other, resulting in a compact crystal packing without any accessible holes, channels or pores (Fig. 6 ▸). To some extent, the complex composition of the title compound expressed by the formula *M* ^II^_3_[*X* ^IV^(OH)_6_][*Y* ^VI^O_4_]_2_·3H_2_O has similarities to those of the mineral thaumasite, Ca_3_[Si(OH)_6_][SO_4_][CO_3_]·12H_2_O (Edge & Taylor, 1971 ▸; Effenberger *et al.*, 1983 ▸), also crystallizing in space group *P*6_3_. In contrast to the title compound, the coordination polyhedron of the earth metal in this mineral is reduced from nine to eight and may be described as a one-face distorted square anti­prism. While the hexa­hydroxidosilicate adopt a similar position as the hexa­hydroxidostannate ion, the two other complex anions of thaumasite are only linked *via* hydrogen bonds to the trimeric units of {CaO_8_} polyhedra as these secondary building units are not cross-linked into layers.

## 4. Synthesis and crystallization

Single crystals of the title compound were obtained in a long-duration experiment exposing a BaSnO_3_ (Celest) pellet prepared by heating equimolar amounts of SnO_2_ and BaO for 40 h at 1688 K to 10 ml of a solution of Na_2_SeO_4_ (Fluka) in a rolled rim glass vessel closed with a snap-on lid. Colorless, hexa­gonal prisms occurred after several months in the sludge of the mouldered BaSnO_3_ pellet.

## 5. Refinement details

Crystal data, data collection and structure refinement details are summarized in Table 4 ▸. All H atoms were clearly identified in difference Fourier syntheses. Their positions were modeled with respect to a common O—H distance of 0.96 Å and a bond angle of 104.9° in case of the water mol­ecule before they were fixed and allowed to ride on the corresponding oxygen atoms. Refinement of two common isotropic temperature factors, one for the hydrogen atoms of the hydroxyl groups and one for the hydrogen atoms of the water mol­ecule, allowed us to check the reliability of their positions.

## Supplementary Material

Crystal structure: contains datablock(s) I. DOI: 10.1107/S2056989022007198/pk2667sup1.cif

Structure factors: contains datablock(s) I. DOI: 10.1107/S2056989022007198/pk2667Isup2.hkl

CCDC reference: 2189782

Additional supporting information:  crystallographic information; 3D view; checkCIF report

## Figures and Tables

**Table 1:** Selected geometric parameters (Å, °)

Se1—O3	1.634 (2)	Se2—O7	1.633 (2)
Se1—O4	1.654 (4)	Se2—O6	1.648 (4)
			
O3^i^—Se1—O3	110.09 (8)	O7^i^—Se2—O7	111.76 (9)
O3—Se1—O4	108.84 (8)	O7—Se2—O6	107.07 (10)

Symmetry code: (i) 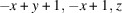 .

**Table 2:** Hydrogen-bond geometry (Å, °)

*D*—H⋯*A*	*D*—H	H⋯*A*	*D*⋯*A*	*D*—H⋯*A*
O1—H1⋯O3	0.96	1.86	2.772 (3)	158
O2—H2⋯O7^ii^	0.96	1.85	2.773 (3)	160
O8—H8*A*⋯O4	0.96	1.82	2.775 (3)	173
O8—H8*B*⋯O7^iii^	0.96	1.98	2.923 (3)	169

Symmetry codes: (ii) 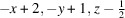 ; (iii) 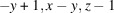 .

**Table 3:** Bond lengths (Å) within the mono-capped {BaO_9_} square anti­prism

Ba1—O3	2.715 (2)	Ba1—O6	2.829 (1)
Ba1—O2	2.737 (2)	Ba1—O8^iv^	2.880 (2)
Ba1—O1^i^	2.777 (2)	Ba1—O8^i^	2.931 (2)
Ba1—O1^ii^	2.779 (2)	Ba1—O8^v^	3.106 (3)
Ba1—O2^iii^	2.782 (2)		

Symmetry codes: (i) *y*, −*x* + *y* + 1, *z* + 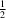 ; (ii) *x* − *y* + 1, *x*, *z* + 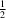 ; (iii) −*x* + *y* + 1, −*x* + 2, *z*; (iv) −*x* + 2, −*y* + 1, *z* + 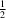 ; (v) −*y* + 1, *x* − *y*, *z*.

**Table 4:** Experimental details

Crystal data	
Chemical formula	Ba_3_[Sn(OH)_6_][SeO_4_]_2_·3H_2_O
*M*_r_	972.73
Crystal system, space group	Hexagonal, *P*6_3_
Temperature (K)	100
*a*, *c* (Å)	9.2550 (6), 11.4441 (8)
*V* (Å^3^)	848.92 (13)
*Z*	2
Radiation type	Mo *K*α
μ (mm^−1^)	12.68
Crystal size (mm)	0.21 × 0.14 × 0.12

Data collection	
Diffractometer	Bruker APEXII CCD
Absorption correction	Multi-scan (*SADABS*; Krause *et al.*, 2015 ▸)
*T*_min_, *T*_max_	0.314, 0.741
No. of measured, independent and observed [*I* > 2σ(*I*)] reflections	112431, 1659, 1653
*R*_int_	0.040
(sin θ/λ)_max_ (Å^−1^)	0.703

Refinement	
*R*[*F* ^2^ > 2σ(*F* ^2^)], *wR*(*F* ^2^), *S*	0.008, 0.018, 1.13
No. of reflections	1659
No. of parameters	74
No. of restraints	1
H-atom treatment	H-atom parameters constrained
Δρ_max_, Δρ_min_ (e Å^−3^)	0.54, −0.38
Absolute structure	Refined as an inversion twin
Absolute structure parameter	0.037 (11)

Computer programs: *APEX2* and *SAINT* (Bruker, 2009 ▸), *SHELXS97* (Sheldrick 2008 ▸), *SHELXL2014/7* (Sheldrick, 2015 ▸), *DIAMOND* (Brandenburg, 2006 ▸), *Mercury* (Macrae *et al.* (2020 ▸) and *publCIF*(Westrip, 2010 ▸).

## supplementary crystallographic information

### Crystal data

**Table:**

Ba_3_[Sn(OH)_6_][SeO_4_]_2_·3H_2_O	*D*_x_ = 3.805 Mg m^−^^3^
*M**_r_* = 972.73	Mo *K*α radiation, λ = 0.71073 Å
Hexagonal, *P*6_3_	Cell parameters from 9633 reflections
*a* = 9.2550 (6) Å	θ = 2.5–30.6°
*c* = 11.4441 (8) Å	µ = 12.68 mm^−^^1^
*V* = 848.92 (13) Å^3^	*T* = 100 K
*Z* = 2	Needle, colourless
*F*(000) = 868	0.21 × 0.14 × 0.12 mm

### Data collection

**Table:**

Bruker APEXII CCD diffractometer	1653 reflections with *I* > 2σ(*I*)
φ and ω scans	*R*_int_ = 0.040
Absorption correction: multi-scan (SADABS; Krause *et al.*, 2015)	θ_max_ = 30.0°, θ_min_ = 2.5°
*T*_min_ = 0.314, *T*_max_ = 0.741	*h* = −13→13
112431 measured reflections	*k* = −13→13
1659 independent reflections	*l* = −16→16

### Refinement

**Table:**

Refinement on *F*^2^	H-atom parameters constrained
Least-squares matrix: full	*w* = 1/[σ^2^(*F*_o_^2^) + (0.0035*P*)^2^ + 1.0003*P*] where *P* = (*F*_o_^2^ + 2*F*_c_^2^)/3
*R*[*F*^2^ > 2σ(*F*^2^)] = 0.008	(Δ/σ)_max_ = 0.001
*wR*(*F*^2^) = 0.018	Δρ_max_ = 0.54 e Å^−^^3^
*S* = 1.13	Δρ_min_ = −0.37 e Å^−^^3^
1659 reflections	Extinction correction: SHELXL-2014/7 (Sheldrick 2015, Fc^*^=kFc[1+0.001xFc^2^λ^3^/sin(2θ)]^-1/4^
74 parameters	Extinction coefficient: 0.00210 (7)
1 restraint	Absolute structure: Refined as an inversion twin.
Primary atom site location: structure-invariant direct methods	Absolute structure parameter: 0.037 (11)
Hydrogen site location: difference Fourier map	

### Special details

**Table:**

Geometry. All esds (except the esd in the dihedral angle between two l.s. planes) are estimated using the full covariance matrix. The cell esds are taken into account individually in the estimation of esds in distances, angles and torsion angles; correlations between esds in cell parameters are only used when they are defined by crystal symmetry. An approximate (isotropic) treatment of cell esds is used for estimating esds involving l.s. planes.
Refinement. Refined as a 2-component inversion twin.

### Fractional atomic coordinates and isotropic or equivalent isotropic displacement parameters (Å^2^)

**Table:**

	*x*	*y*	*z*	*U*_iso_*/*U*_eq_	
Ba1	0.85513 (2)	0.67674 (2)	0.76533 (2)	0.00444 (4)	
Sn1	1.0000	1.0000	0.51521 (5)	0.00320 (5)	
O1	0.9097 (3)	0.7983 (3)	0.4052 (2)	0.0057 (4)	
H1	0.8736	0.6914	0.4403	0.030 (7)*	
O2	1.1069 (3)	0.9077 (3)	0.6279 (2)	0.0056 (4)	
H2	1.1548	0.8586	0.5787	0.030 (7)*	
Se1	0.6667	0.3333	0.50722 (5)	0.00388 (9)	
Se2	0.6667	0.3333	0.96470 (5)	0.00512 (9)	
O6	0.6667	0.3333	0.8207 (3)	0.0072 (7)	
O4	0.6667	0.3333	0.3627 (4)	0.0076 (6)	
O3	0.7824 (3)	0.5248 (3)	0.55332 (19)	0.0108 (4)	
O7	0.6848 (3)	0.1745 (3)	1.0066 (2)	0.0106 (4)	
O8	0.9600 (3)	0.4949 (2)	0.23440 (18)	0.0095 (4)	
H8A	0.8628	0.4361	0.2827	0.054 (11)*	
H8B	0.9175	0.4875	0.1568	0.054 (11)*	

### Atomic displacement parameters (Å^2^)

**Table:**

	*U*^11^	*U*^22^	*U*^33^	*U*^12^	*U*^13^	*U*^23^
Ba1	0.00500 (6)	0.00347 (6)	0.00436 (6)	0.00175 (5)	−0.00001 (8)	−0.00006 (8)
Sn1	0.00351 (7)	0.00351 (7)	0.00258 (10)	0.00175 (3)	0.000	0.000
O1	0.0070 (11)	0.0036 (11)	0.0054 (10)	0.0018 (9)	0.0002 (8)	−0.0001 (8)
O2	0.0062 (11)	0.0066 (11)	0.0057 (10)	0.0044 (10)	0.0001 (8)	0.0009 (8)
Se1	0.00407 (14)	0.00407 (14)	0.0035 (2)	0.00204 (7)	0.000	0.000
Se2	0.00486 (14)	0.00486 (14)	0.0057 (2)	0.00243 (7)	0.000	0.000
O6	0.0085 (10)	0.0085 (10)	0.0046 (17)	0.0043 (5)	0.000	0.000
O4	0.0103 (10)	0.0103 (10)	0.0023 (13)	0.0051 (5)	0.000	0.000
O3	0.0137 (11)	0.0049 (10)	0.0087 (9)	0.0008 (8)	−0.0013 (8)	−0.0022 (8)
O7	0.0133 (11)	0.0089 (10)	0.0125 (9)	0.0077 (9)	−0.0017 (9)	0.0024 (8)
O8	0.0071 (8)	0.0113 (9)	0.0096 (10)	0.0041 (7)	0.0001 (7)	0.0006 (7)

### Geometric parameters (Å, º)

**Table:**

Ba1—O3	2.715 (2)	O1—Ba1^ix^	2.777 (2)
Ba1—O2	2.737 (2)	O1—Ba1^viii^	2.779 (2)
Ba1—O1^i^	2.777 (2)	O1—H1	0.9600
Ba1—O1^ii^	2.779 (2)	O2—Ba1^vii^	2.782 (2)
Ba1—O2^iii^	2.782 (2)	O2—H2	0.9600
Ba1—O6	2.829 (1)	Se1—O3^xi^	1.633 (2)
Ba1—O8^iv^	2.880 (2)	Se1—O3	1.634 (2)
Ba1—O8^i^	2.931 (2)	Se1—O3^v^	1.634 (2)
Ba1—O7^v^	3.106 (3)	Se1—O4	1.654 (4)
Ba1—Se2	3.5786 (4)	Se2—O7^xi^	1.633 (2)
Ba1—Sn1^vi^	3.8620 (5)	Se2—O7^v^	1.633 (2)
Ba1—Sn1	3.8639 (5)	Se2—O7	1.633 (2)
Sn1—O1^iii^	2.052 (2)	Se2—O6	1.648 (4)
Sn1—O1	2.052 (2)	Se2—Ba1^xi^	3.5786 (4)
Sn1—O1^vii^	2.052 (2)	Se2—Ba1^v^	3.5786 (4)
Sn1—O2	2.054 (2)	O6—Ba1^xi^	2.8288 (8)
Sn1—O2^vii^	2.054 (2)	O6—Ba1^v^	2.8288 (8)
Sn1—O2^iii^	2.054 (2)	O7—Ba1^xi^	3.106 (3)
Sn1—Ba1^viii^	3.8620 (5)	O8—Ba1^xii^	2.880 (2)
Sn1—Ba1^ix^	3.8620 (5)	O8—Ba1^ix^	2.931 (2)
Sn1—Ba1^x^	3.8620 (5)	O8—H8A	0.9600
Sn1—Ba1^vii^	3.8639 (5)	O8—H8B	0.9600
Sn1—Ba1^iii^	3.8639 (5)		
			
O3—Ba1—O2	77.58 (7)	Ba1^viii^—Sn1—Ba1^ix^	71.188 (11)
O3—Ba1—O1^i^	142.66 (7)	O1^iii^—Sn1—Ba1^x^	43.99 (7)
O2—Ba1—O1^i^	99.65 (6)	O1—Sn1—Ba1^x^	94.36 (7)
O3—Ba1—O1^ii^	144.19 (7)	O1^vii^—Sn1—Ba1^x^	43.93 (7)
O2—Ba1—O1^ii^	70.22 (5)	O2—Sn1—Ba1^x^	137.27 (7)
O1^i^—Ba1—O1^ii^	60.66 (10)	O2^vii^—Sn1—Ba1^x^	86.69 (7)
O3—Ba1—O2^iii^	77.32 (7)	O2^iii^—Sn1—Ba1^x^	135.94 (6)
O2—Ba1—O2^iii^	60.20 (10)	Ba1^viii^—Sn1—Ba1^x^	71.188 (11)
O1^i^—Ba1—O2^iii^	69.60 (5)	Ba1^ix^—Sn1—Ba1^x^	71.188 (11)
O1^ii^—Ba1—O2^iii^	99.11 (6)	O1^iii^—Sn1—Ba1	136.01 (7)
O3—Ba1—O6	76.38 (9)	O1—Sn1—Ba1	85.66 (7)
O2—Ba1—O6	144.30 (7)	O1^vii^—Sn1—Ba1	136.07 (7)
O1^i^—Ba1—O6	115.84 (7)	O2—Sn1—Ba1	42.73 (7)
O1^ii^—Ba1—O6	123.39 (8)	O2^vii^—Sn1—Ba1	93.29 (7)
O2^iii^—Ba1—O6	134.70 (7)	O2^iii^—Sn1—Ba1	44.06 (6)
O3—Ba1—O8^iv^	70.52 (6)	Ba1^viii^—Sn1—Ba1	108.832 (5)
O2—Ba1—O8^iv^	81.46 (7)	Ba1^ix^—Sn1—Ba1	108.833 (5)
O1^i^—Ba1—O8^iv^	146.58 (6)	Ba1^x^—Sn1—Ba1	179.974 (12)
O1^ii^—Ba1—O8^iv^	89.23 (6)	O1^iii^—Sn1—Ba1^vii^	136.08 (7)
O2^iii^—Ba1—O8^iv^	134.31 (6)	O1—Sn1—Ba1^vii^	136.01 (7)
O6—Ba1—O8^iv^	67.08 (4)	O1^vii^—Sn1—Ba1^vii^	85.66 (7)
O3—Ba1—O8^i^	74.22 (6)	O2—Sn1—Ba1^vii^	44.06 (6)
O2—Ba1—O8^i^	127.90 (6)	O2^vii^—Sn1—Ba1^vii^	42.73 (7)
O1^i^—Ba1—O8^i^	79.11 (7)	O2^iii^—Sn1—Ba1^vii^	93.29 (7)
O1^ii^—Ba1—O8^i^	139.10 (6)	Ba1^viii^—Sn1—Ba1^vii^	179.974 (12)
O2^iii^—Ba1—O8^i^	71.31 (7)	Ba1^ix^—Sn1—Ba1^vii^	108.832 (5)
O6—Ba1—O8^i^	66.39 (4)	Ba1^x^—Sn1—Ba1^vii^	108.832 (5)
O8^iv^—Ba1—O8^i^	126.49 (7)	Ba1—Sn1—Ba1^vii^	71.147 (11)
O3—Ba1—O7^v^	126.88 (6)	O1^iii^—Sn1—Ba1^iii^	85.66 (7)
O2—Ba1—O7^v^	136.68 (7)	O1—Sn1—Ba1^iii^	136.07 (7)
O1^i^—Ba1—O7^v^	80.61 (7)	O1^vii^—Sn1—Ba1^iii^	136.01 (7)
O1^ii^—Ba1—O7^v^	72.68 (6)	O2—Sn1—Ba1^iii^	93.29 (7)
O2^iii^—Ba1—O7^v^	148.89 (7)	O2^vii^—Sn1—Ba1^iii^	44.06 (6)
O6—Ba1—O7^v^	52.54 (8)	O2^iii^—Sn1—Ba1^iii^	42.73 (7)
O8^iv^—Ba1—O7^v^	76.42 (6)	Ba1^viii^—Sn1—Ba1^iii^	108.832 (5)
O8^i^—Ba1—O7^v^	94.97 (6)	Ba1^ix^—Sn1—Ba1^iii^	179.974 (12)
O3—Ba1—Se2	103.02 (5)	Ba1^x^—Sn1—Ba1^iii^	108.832 (5)
O2—Ba1—Se2	155.03 (5)	Ba1—Sn1—Ba1^iii^	71.147 (11)
O1^i^—Ba1—Se2	94.54 (5)	Ba1^vii^—Sn1—Ba1^iii^	71.147 (11)
O1^ii^—Ba1—Se2	99.78 (5)	Sn1—O1—Ba1^ix^	105.23 (9)
O2^iii^—Ba1—Se2	144.71 (5)	Sn1—O1—Ba1^viii^	105.15 (10)
O6—Ba1—Se2	26.66 (7)	Ba1^ix^—O1—Ba1^viii^	108.03 (8)
O8^iv^—Ba1—Se2	75.44 (4)	Sn1—O1—H1	117.0
O8^i^—Ba1—Se2	74.87 (4)	Ba1^ix^—O1—H1	104.7
O7^v^—Ba1—Se2	27.11 (4)	Ba1^viii^—O1—H1	115.9
O3—Ba1—Sn1^vi^	164.39 (5)	Sn1—O2—Ba1	106.66 (10)
O2—Ba1—Sn1^vi^	89.44 (5)	Sn1—O2—Ba1^vii^	105.06 (9)
O1^i^—Ba1—Sn1^vi^	30.84 (5)	Ba1—O2—Ba1^vii^	109.09 (8)
O1^ii^—Ba1—Sn1^vi^	30.85 (5)	Sn1—O2—H2	105.1
O2^iii^—Ba1—Sn1^vi^	88.77 (5)	Ba1—O2—H2	112.5
O6—Ba1—Sn1^vi^	118.99 (7)	Ba1^vii^—O2—H2	117.6
O8^iv^—Ba1—Sn1^vi^	116.51 (4)	O3^xi^—Se1—O3	110.09 (8)
O8^i^—Ba1—Sn1^vi^	108.24 (4)	O3^xi^—Se1—O3^v^	110.09 (8)
O7^v^—Ba1—Sn1^vi^	68.67 (4)	O3—Se1—O3^v^	110.09 (8)
Se2—Ba1—Sn1^vi^	92.415 (12)	O3^xi^—Se1—O4	108.84 (8)
O3—Ba1—Sn1	68.85 (5)	O3—Se1—O4	108.84 (8)
O2—Ba1—Sn1	30.61 (5)	O3^v^—Se1—O4	108.84 (8)
O1^i^—Ba1—Sn1	89.73 (5)	O7^xi^—Se2—O7^v^	111.76 (9)
O1^ii^—Ba1—Sn1	89.69 (5)	O7^xi^—Se2—O7	111.76 (9)
O2^iii^—Ba1—Sn1	30.89 (5)	O7^v^—Se2—O7	111.76 (9)
O6—Ba1—Sn1	144.71 (7)	O7^xi^—Se2—O6	107.07 (10)
O8^iv^—Ba1—Sn1	105.32 (4)	O7^v^—Se2—O6	107.07 (10)
O8^i^—Ba1—Sn1	97.70 (4)	O7—Se2—O6	107.07 (10)
O7^v^—Ba1—Sn1	162.33 (4)	O7^xi^—Se2—Ba1^xi^	139.72 (9)
Se2—Ba1—Sn1	170.516 (10)	O7^v^—Se2—Ba1^xi^	107.28 (9)
Sn1^vi^—Ba1—Sn1	95.571 (6)	O7—Se2—Ba1^xi^	60.11 (9)
O1^iii^—Sn1—O1	86.27 (10)	O6—Se2—Ba1^xi^	50.387 (8)
O1^iii^—Sn1—O1^vii^	86.27 (10)	O7^xi^—Se2—Ba1^v^	60.11 (9)
O1—Sn1—O1^vii^	86.27 (10)	O7^v^—Se2—Ba1^v^	139.72 (9)
O1^iii^—Sn1—O2	178.66 (12)	O7—Se2—Ba1^v^	107.28 (9)
O1—Sn1—O2	93.93 (9)	O6—Se2—Ba1^v^	50.387 (8)
O1^vii^—Sn1—O2	95.06 (9)	Ba1^xi^—Se2—Ba1^v^	83.696 (12)
O1^iii^—Sn1—O2^vii^	95.07 (9)	O7^xi^—Se2—Ba1	107.28 (9)
O1—Sn1—O2^vii^	178.66 (12)	O7^v^—Se2—Ba1	60.11 (9)
O1^vii^—Sn1—O2^vii^	93.93 (9)	O7—Se2—Ba1	139.72 (9)
O2—Sn1—O2^vii^	84.73 (10)	O6—Se2—Ba1	50.387 (8)
O1^iii^—Sn1—O2^iii^	93.93 (9)	Ba1^xi^—Se2—Ba1	83.697 (12)
O1—Sn1—O2^iii^	95.06 (9)	Ba1^v^—Se2—Ba1	83.696 (12)
O1^vii^—Sn1—O2^iii^	178.66 (12)	Se2—O6—Ba1^xi^	102.95 (7)
O2—Sn1—O2^iii^	84.73 (10)	Se2—O6—Ba1^v^	102.95 (7)
O2^vii^—Sn1—O2^iii^	84.73 (10)	Ba1^xi^—O6—Ba1^v^	115.13 (5)
O1^iii^—Sn1—Ba1^viii^	43.93 (7)	Se2—O6—Ba1	102.95 (7)
O1—Sn1—Ba1^viii^	44.00 (7)	Ba1^xi^—O6—Ba1	115.13 (5)
O1^vii^—Sn1—Ba1^viii^	94.36 (7)	Ba1^v^—O6—Ba1	115.13 (5)
O2—Sn1—Ba1^viii^	135.94 (6)	Se1—O3—Ba1	135.14 (12)
O2^vii^—Sn1—Ba1^viii^	137.27 (7)	Se2—O7—Ba1^xi^	92.78 (11)
O2^iii^—Sn1—Ba1^viii^	86.69 (7)	Ba1^xii^—O8—Ba1^ix^	110.53 (7)
O1^iii^—Sn1—Ba1^ix^	94.36 (7)	Ba1^xii^—O8—H8A	105.3
O1—Sn1—Ba1^ix^	43.93 (7)	Ba1^ix^—O8—H8A	119.5
O1^vii^—Sn1—Ba1^ix^	43.99 (7)	Ba1^xii^—O8—H8B	114.2
O2—Sn1—Ba1^ix^	86.69 (7)	Ba1^ix^—O8—H8B	102.6
O2^vii^—Sn1—Ba1^ix^	135.94 (6)	H8A—O8—H8B	105.0
O2^iii^—Sn1—Ba1^ix^	137.27 (7)		

Symmetry codes: (i) *y*, −*x*+*y*+1, *z*+1/2; (ii) *x*−*y*+1, *x*, *z*+1/2; (iii) −*x*+*y*+1, −*x*+2, *z*; (iv) −*x*+2, −*y*+1, *z*+1/2; (v) −*y*+1, *x*−*y*, *z*; (vi) −*x*+2, −*y*+2, *z*+1/2; (vii) −*y*+2, *x*−*y*+1, *z*; (viii) *y*, −*x*+*y*+1, *z*−1/2; (ix) *x*−*y*+1, *x*, *z*−1/2; (x) −*x*+2, −*y*+2, *z*−1/2; (xi) −*x*+*y*+1, −*x*+1, *z*; (xii) −*x*+2, −*y*+1, *z*−1/2.

### Hydrogen-bond geometry (Å, º)

**Table:**

*D*—H···*A*	*D*—H	H···*A*	*D*···*A*	*D*—H···*A*
O1—H1···O3	0.96	1.86	2.772 (3)	158
O2—H2···O7^xii^	0.96	1.85	2.773 (3)	160
O8—H8*A*···O4	0.96	1.82	2.775 (3)	173
O8—H8*B*···O7^xiii^	0.96	1.98	2.923 (3)	169

Symmetry codes: (xii) −*x*+2, −*y*+1, *z*−1/2; (xiii) −*y*+1, *x*−*y*, *z*−1.

## References

[bb1] Andara, A., Salvado, M. A., Fernández-González, Á., García-Granda, S. & Prieto, M. (2005). *Z. Kristallogr. New Cryst. Struct.* **220**, 5–6.

[bb2] Basciano, L. C., Peterson, R. C., Roeder, P. L. & Swainson, I. (1998). *Can. Mineral.* **36**, 1203–1210.

[bb3] Brandenburg, K. (2006). *DIAMOND*. Crystal Impact GbR, Bonn, Germany.

[bb4] Bruker (2009). *APEX2*, *SADABS* and *SAINT*. Bruker AXS Inc., Madison, Wisconsin, USA.

[bb5] Edge, R. A. & Taylor, H. F. W. (1971). *Acta Cryst.* B**27**, 594–601.

[bb6] Effenberger, H., Kirfel, A., Will, G. & Zobetz, E. (1983). *Neues Jahrb. Mineral. Monatsh.* pp. 60–68.

[bb7] Fortes, A. D., Alfè, D., Hernández, E. R. & Gutmann, M. J. (2015). *Acta Cryst.* B**71**, 313–327. 10.1107/S2052520615006824 PMC4450603 26027007

[bb8] Fortes, A. D. & Gutmann, M. J. (2014). *Acta Cryst.* E**70**, 134–137. 10.1107/S1600536814018698 PMC4186159 25309161

[bb9] Kamaha, S. & Reuter, H. (2009). *Z. Anorg. Allg. Chem.* **635**, 2058–2064.

[bb10] Kolitsch, U. (2002). *Acta Cryst.* E**58**, i3–i5.

[bb11] Krause, L., Herbst-Irmer, R., Sheldrick, G. M. & Stalke, D. (2015). *J. Appl. Cryst.* **48**, 3–10. 10.1107/S1600576714022985 PMC4453166 26089746

[bb12] Macrae, C. F., Sovago, I., Cottrell, S. J., Galek, P. T. A., McCabe, P., Pidcock, E., Platings, M., Shields, G. P., Stevens, J. S., Towler, M. & Wood, P. A. (2020). *J. Appl. Cryst.* **53**, 226–235. 10.1107/S1600576719014092 PMC6998782 32047413

[bb13] Scholder, R. (1981). *Hydroxosalze* in *Handbuch der Präparativen Anorganischen Chemie*, edited by G. Brauer, p. 1771.Stuttgart: Enke.

[bb14] Sheldrick, G. M. (2008). *Acta Cryst.* A**64**, 112–122. 10.1107/S0108767307043930 18156677

[bb15] Sheldrick, G. M. (2015). *Acta Cryst.* C**71**, 3–8.

[bb16] Strunz, H. & Nickel, E. H. (1998). *Strunz Mineralogical Tables, Chemical–Structural Mineral Classification System*, 9th ed., pp. 232–233. Stuttgart: Schweizerbart.

[bb17] Weil, M. & Bonneau, B. (2014). *Acta Cryst.* E**70**, 54–57. 10.1107/S1600536814011799 PMC4158548 25249853

[bb18] Westrip, S. P. (2010). *J. Appl. Cryst.* **43**, 920–925.

